# “Après Mois, Le Déluge”: Preparing for the Coming Data Flood in the MRI-Guided Radiotherapy Era

**DOI:** 10.3389/fonc.2019.00983

**Published:** 2019-09-30

**Authors:** Kendall J. Kiser, Benjamin D. Smith, Jihong Wang, Clifton D. Fuller

**Affiliations:** ^1^John P. and Kathrine G. McGovern Medical School, University of Texas Health Science Center, Houston, TX, United States; ^2^School of Biomedical Informatics, University of Texas Health Science Center, Houston, TX, United States; ^3^Department of Radiation Oncology, University of Texas MD Anderson Cancer Center, Houston, TX, United States; ^4^Department of Radiation Physics, University of Texas MD Anderson Cancer Center, Houston, TX, United States

**Keywords:** MRI, MRI-guided radiotherapy, MR LINAC, informatics, biomedical informatics, clinical informatics, imaging informatics, radiomics

## Abstract

Magnetic resonance imaging provides a sea of quantitative and semi-quantitative data. While radiation oncologists already navigate a pool of clinical (semantic) and imaging data, the tide will swell with the advent of hybrid MRI/linear accelerator devices and increasing interest in MRI-guided radiotherapy (MRIgRT), including adaptive MRIgRT. The variety of MR sequences (of greater complexity than the single parameter Hounsfield unit of CT scanning routinely used in radiotherapy), the workflow of adaptive fractionation, and the sheer quantity of daily images acquired are challenges for scaling this technology. Biomedical informatics, which is the science of information in biomedicine, can provide helpful insights for this looming transition. Funneling MRIgRT data into clinically meaningful information streams requires committing to the flow of inter-institutional data accessibility and interoperability initiatives, standardizing MRIgRT dosimetry methods, streamlining MR linear accelerator workflow, and standardizing MRI acquisition and post-processing. This review will attempt to conceptually ford these topics using clinical informatics approaches as a theoretical bridge.

## Introduction

Use of magnetic resonance imaging (MRI) rather than computed tomography (CT) for radiotherapy (RT) planning can be highly desirable because MRI visualizes soft tissues with superior contrast and resolution (1), introduces unique sequences and contrast agents for delineating specific tumors and anatomic subsites (1, 2), and permits daily adaptive radiotherapy (ART) without added CT radiation dose (3–5). MRI-guided ART (MRIgART) machines have advanced from low-field (0.35 Tesla) magnets with Cobalt-60 radiation sources (6) to diagnostic-strength magnetic fields (1.5 Tesla) fully integrated with linear accelerators (7, 8) in <5 years. Over the coming decade, MRI-guided RT (MRIgRT) may change clinical practice paradigms (9). The earliest adopter of MRIgART, Washington University in St. Louis (WUSTL), has already altered its management of breast and abdominal malignancies (10). However, to scale MRIgRT, workflow and standardization challenges that do not exist in CT-guided planning need be resolved.

First, MR scan reproducibility is more complicated than for CT. Consider a T1-weighted scan: pixel intensities are predominately derived from longitudinal relaxation time (T1), an intrinsic tissue property. Nevertheless, proton density (H) and transverse relaxation time (T2) (which are also intrinsic tissue properties) may greatly influence overall signal intensity (11) depending on repetition time (TR) and echo time (TE) parameters (see Equation 1).

(1) $$\begin{array}{r} S=K\cdot \left[H\right]\cdot \left(1-e^{-\frac{TR}{T1}}\right)\cdot e^{-\frac{TE}{T2}}\; \end{array}$$

These parameters are not standardized across institutions or vendors, so a T1-weighted scan acquired by a given vendor's machine is not necessarily equivalent in terms of observed intensity as one acquired by another manufacturer. Similarly, MRI acquisition suffers from geometric distortions that are model-, vendor-, software-, shim-, and coil-dependent. Proper correction also depends on variable user-driven acquisition parameters (12, 13).

**Figure 1:**
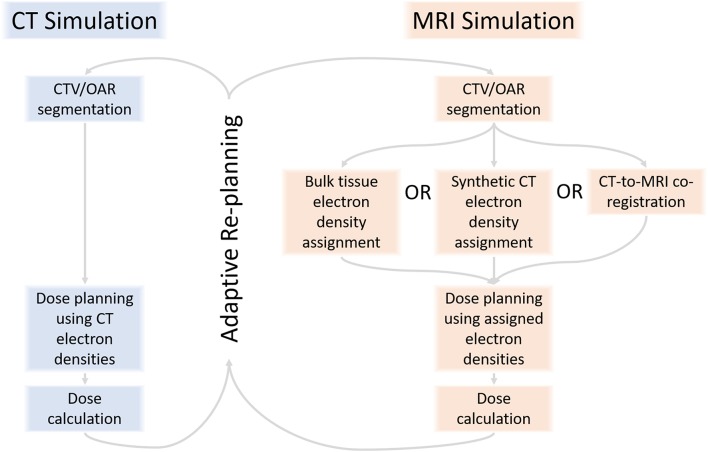
RT planning with MRI requires assignment of electron density to pixels or tissues to calculate dose, a step that is not part of CT-guided RT planning.

Second, there are more steps in MRIgRT planning than CT-guided planning. MRI does not convey electron density information necessary for standard photon dosimetry, so either (1) MRI data must be registered to CT Hounsfield unit values (14–17), or (2) a synthetic CT (sCT) must be algorithmically generated from MRI (18, 19), or (3) tissue types must be assigned a single, indiscriminate density (18) (Figure 1). Additionally, MRIgART fractionation requires far more time of the patient, radiation oncologist, and staff than traditional RT treatment courses.

**Figure 2:**
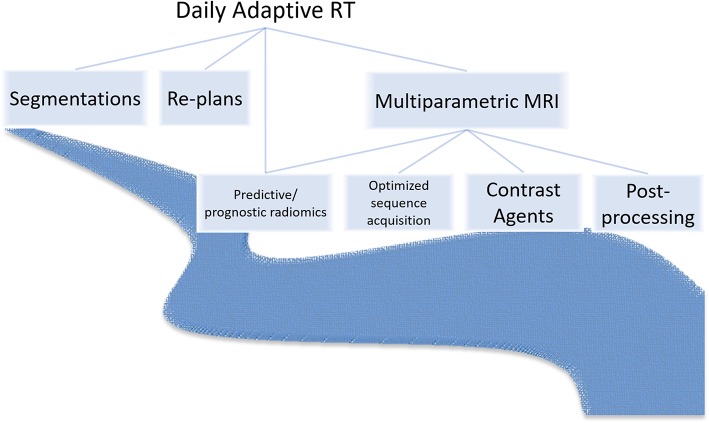
MRI-guided radiotherapy may introduce a deluge of new image sequences, optimization needs, image post-processing needs, contrast agents, prognostic and predictive radiomics features, and adaptive imaging and clinical data.

Third, RT generates seas of imaging data (20, 21) and structured and unstructured clinical data (22–24) that will deepen with multiparametric MRI sequences, unique contrast agents, and radiomics features and MRIgART daily images, contours, and plans (Figure 2). At our institution, MRIgRT generates roughly four times as many bytes of data as CT-guided RT (1 Gb per patient per day vs. 250 Mb). *Not all data are fit for clinical decision-making or scientific inquiry*. For example, MRIgART could quantitatively track soft tissue tumor shrinkage, but the results would only be clinically actionable if the segmentation method were systematic and reproduceable. Interpretability and reproducibility of MRI data across institutions and vendors is not a given.

Effective use of “biomedical data, information, and knowledge for scientific inquiry, problem-solving and decision making” formally defines the field of biomedical informatics (BMI) (25). The raison d'être of BMI is to reduce data (which are meaningless symbols) into information (which is data plus meaning), and further into knowledge (which is information that is justifiably believed to be true) (26). This paper considers BMI concepts in the context of scaling MRIgRT (see Table 1) and critiques existing literature from the perspective of how it increases information and knowledge to streamline MRIgRT workflow and ensure the consistency and usability of MRIgRT data.

**Table 1:** Biomedical informatics concepts.

**Concept**	**Explanation**	**Example**
Data, Information, Knowledge, Wisdom (DIKW) pyramid	Data, information, and knowledge are not synonymous terms. Information is data plus meaning. Knowledge is information plus a justifiable belief in its veracity. Some models also include wisdom as a tier above knowledge (27), but Bernstam et al. (26) omit this.	Radiation oncologist expert interpretation refines dose-volume histogram (DVH) data into information. Statistical analyses then extract and justify knowledge.
The cycle of clinical information flow	Clinical care generates data, which are used in biomedical research, the results of which develop prevention and treatment standards, which are built into clinical protocols, which are built into clinical decision support and order-entry systems, which directly influence clinical care, etc. (28).	MRIgRT outcomes data are recorded in electronic health records, then leveraged in clinical research, which begins to establish the role of MRIgRT, which dictates clinical protocols, which are built into clinical decision support and other health information technology systems, which collaborate with physicians during MRIgRT treatment evaluation and planning, etc.
Data standards	Data standards define and describe “common and repeated use, rules, guidelines or characteristics for activities or their results, aimed at the optimum degree of order” (29).	Digital Imaging and Communications (DICOM), DICOM-RT, Fast Healthcare Interoperable Resources (FHIR), AAPM Task Group 263 consensus nomenclature for dosimetric structures (24).
Interoperability	Interoperability is “the ability of a system or product to work with other systems or products without special effort on the part of the customer” (30). Data standards precede interoperability.	FHIR-conforming electronic health record applications are interoperable between different vendors that also conform with FHIR.
Consumer health informatics	Consumer health informatics is a subfield of biomedical informatics focused on the interactions of patients and consumers with health information systems, catalyzed by mobile technologies and the Internet (31).	Patients log acute and late toxicities during and after MRIgRT in applications built for their phones.

## How Is Biomedical Informatics Relevant to MRIgRT Currently?

MRI is already an established modality for image-guided RT of nasopharynx, brain, spine, liver, pancreas, prostate, and female genital tract cancers (1, 2, 32). In each case, standardization preserves the integrity of critical decision-making information. Consider MRIgRT for prostate cancer (33, 34). Radiology standards exist for MRI acquisition, interpretation, and reporting (35). These improve reporting among radiologists of varying experience levels (36), lest anatomic delineation suffer poor consistency and patient outcomes comparison data be meaningless. At the MR-CT co-registration step, co-registration between limited field-of-view images is the recommended standard because error is increased when the field-of-view includes the anatomically variable bladder and rectum (37). At the RT planning step, guidelines from the European Society for Radiotherapy and Oncology (ESTRO) (38) and Radiation Therapy Oncology Group (RTOG) (39) standardize MRI-based clinical target and organ-at-risk (OAR) contour volumes. Ostensibly, these steps culminate in more conformal prostate RT, but MRIgRT has proved only modest decreases in OAR toxicity compared to CT-guided RT (40, 41), especially with the development of rectal spacer hydrogel (42). Evaluating data quality and the assumptions used to establish the clinical value of MRIgRT will be a critical BMI task in the coming decade, one that should exploit emerging consumer health informatics approaches.

### BMI Considerations for MRIgRT Dosimetry

**Figure 3:**
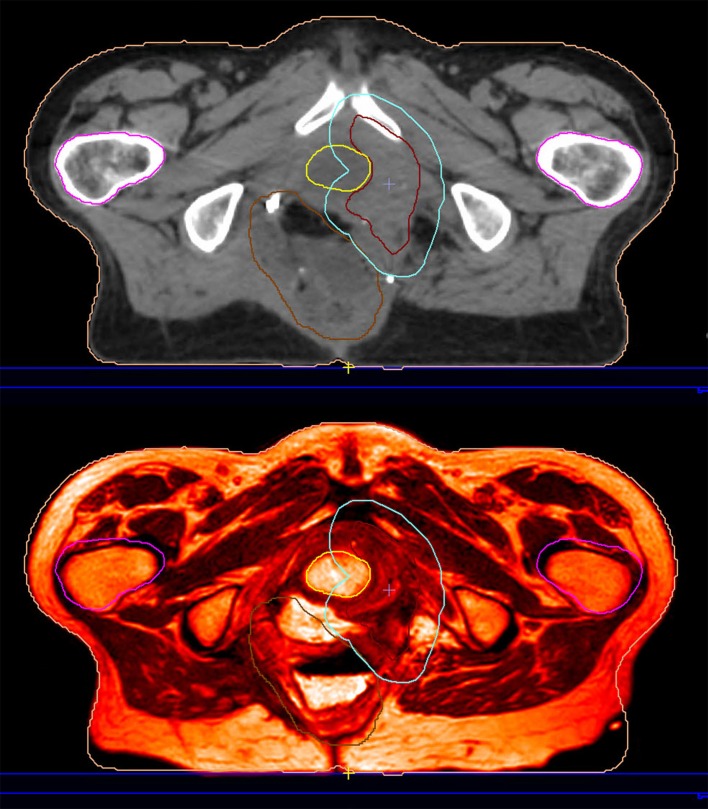
This patient was reirradiated for a rectal adenocarcinoma recurrence on a hybrid magnetic resonance-guided linear accelerator. The planning image **(top)** is an electron density imputation based on the simulation MRI **(bottom)**.

As already noted, MRIgRT requires either MRI-CT co-registration, sCT generation, or bulk density assignment to calculate tissue radiation dose. MR-only workflows employ either the second or third approach (with the caveat that atlas-based sCT generation techniques employ MRI-CT co-registration to generate an MRI atlas). Improving MR-only RT is strongly motivated by the desire to simplify adaptive workflow for integrated magnetic resonance linear accelerators (MRLs). Figure 3 exemplifies an imputed electron density map in a patient treated on an MRL. We refer the reader to Table 1 in (34) for an overview of current MRL platforms.

#### Bulk Density Override and Synthetic CT

Homogenous bulk density override is crude but achieves reasonable dosimetric accuracy if specific structures (e.g., cortical bone) are contoured by a radiation oncologist and separately assigned a unique density (43–45). In contrast, sCT generation by voxel-based or atlas-based methods obviates the need for time-intensive contouring and therefore may be preferred. Johnstone et al. extensively discussed sCT generation methods in a systematic review (18). Many sCT results appear clinically comparable to CT. In the brain, sCT-derived digitally reconstructed radiographs were as geometrically robust as those derived from CT (46). In the prostate, sCT gamma passing rates have been comparable with CT gamma passing rates (median 1%/1 mm pass rate of 100% for almost all regions of interest across 29 patient scans) (47). Nevertheless, MR-only workflow introduces unique BMI considerations. For example, prostate RT plans are more precise with setup to intraprostatic gold or titanium fiducial implants (48), but these are visualized as signal void on conventional MRI sequences and poorly differentiated from calcifications. Maspero et al. (49) reported that 3/48 fiducial implants were imprecisely and inaccurately identified by five radiation technologists when visualized only on MRI. On the other hand, new setup techniques based on MR daily imaging might obviate the need for fiducials. Thoughtful consideration of parameters like these are needed to ensure not only the safety of the method but the quality and reproducibility of the data. Consensus is also needed to establish the standard metrics by which sCT quality should be gauged (18).

#### MRI-CT Co-registration

MRIs can be registered to CT rigidly (without warping the MRI) or by a deformation vector field. Deformable registration confers a more concordant result than rigid registration between diagnostic CT and simulation CT (50–53), but recent work from our group did not demonstrate the same advantage between simulation MRI and simulation CT, at least in the head and neck (17). This should not imply that rigid registration is adequately accurate, since we also found that the registration error (whether by deformable or rigid means) may not be within the target tolerance recommended by the American Association of Physicists in Medicine (AAPM) Task Group 132 (Dice similarity coefficient > 0.8) (54). Perhaps registration was poor because of MR geometric distortion, or perhaps because not all OARs are clearly delineated on both CT and MRI. Regardless, this informs our view that sCT may be preferred to CT-MRI co-registration for RT dose deposition calculation, pending needed standardizations as discussed above.

## Workflow Considerations for Integrated MR Linear Accelerators

The “holy grail” of MRL RT is to see the target at setup, adapt the plan as needed, and gate by watching anatomic movement while the beam is on. The experience of the Department of Radiation Oncology at WUSTL, which introduced the first 0.35 T tri-cobalt-60 MRIgRT system (ViewRay, Oakwood Village, OH, USA) in the USA (6), provides great insight into adaptive MRL clinical informatics challenges. In a Phase I trial intended to demonstrate the temporal feasibility of MRI-guided stereotactic body radiation (SBRT), median on-table time per fraction was 79 min and consisted of MR set-up, physician arrival, patient localization, re-segmentation, re-planning, quality assurance (QA), and beam on-time (3). Almost all fractions (81/97) were adapted based on the patient's anatomy-of-day to avoid irradiating OARs. Despite fear that patients would not tolerate fractions longer than 80 min, all 20 patients completed their treatments as prescribed.

MRL RT has evolved into a dominant indication for abdominal and breast cancers at WUSTL, primarily because motion gating and daily adaptation prevent OAR dose constraint violations (10). The MRL has also prevented violations in hypofractionated lung tumor stereotactic radiotherapy, and enabled adaptive GTV reductions by as much as 65% (55). However, adaptation remains time-intensive. Current systems require physician attendance during every fraction (56), which would not be sustainable at sites that lack sufficient physician and support staff. Three studies from the University of Alberta examined whether automated ROI segmentation can decrease the burden on physician time. In the first, a pulse-coupled neural network (PCNN) was developed to segment lung tumors in the context of adaptive MRL RT (57). The PCNN achieved a strong Dice Similarity Index (DSI) of 0.87–0.92, but it required training on a unique dataset of manually-generated contours per patient. A follow-up study improved DSI (58) with a pre-segmentation deformable registration methodology, but still required a physician to segment lung tumor across multiple image frames. In the third study, DSI and other conformality metrics improved using a fully convolutional neural network (FCNN), but the FCNN still needed to be trained on 30 manual contours per patient. While these studies demonstrate that automated segmentations of lung tumors for MRL RT can achieve high fidelity, they may not hasten adaptive, online MRL workflow. In contrast, a WUSTL novel tri-convolutional neural network architecture capable of segmenting liver, kidneys, stomach, bowel, and duodenum did reduce manual segmentation time by 75% at WUSTL (59).

Intra and inter-observer variation in segmentation quality has been documented using many imaging modalities in pelvic (60, 61), lung (62), breast (63), head-and-neck (60, 64), and brain (65) RT planning. In the specific context of MRL for lung stereotactic body RT, Wee et al. (66) found no significant intra or inter-observer variation in manual segmentations of images acquired on a 0.35 T MRL. However, only two radiation oncologist observers were compared, for only one ROI (gross tumor volume) (66), limiting the generalizability of the study conclusion.

To hasten MRL re-planning, WUSTL simplified the number of planning objectives by grouping OAR structures into a single structure (67). This both increased PTV coverage and simplified re-planning by reducing the computational burden of satisfying a greater number of competing objectives. This work was specific to pancreatic cancer planning objectives, but the approach may be amenable to re-planning for other sites.

Intrafraction motion management/gating is a hotly anticipated MRL advantage. Han et al. (68) applied 3D-Rotating Cartesian K-space MRI (4D-ROCK-MRI) in an MRL RT workflow to improve lung tumor motion tracking. 4D-ROCK-MRI improved image quality and motion tracking and decreased lung cancer GTV variability compared with 4D-CT, which suffers from 2D-slice “stitching” artifact. The authors reason that it might capture motion better than 2D-CINE MRI because it acquires data over a 7 min interval, while the latter screens less than a minute of data. Cusumano et al. (69) compared 4D-CT and 2D-cine MR motion data acquired at the time of simulation with complete 2D-cine MR datasets acquired over entire MRIgRT treatment courses. Simulation 2D-cine MR appeared better than simulation 4D-CT, though not significantly. Patients with large motion amplitudes at the time of simulation tended to have more variable amplitudes throughout their treatment course, but even targets with steady amplitudes frequently drifted from the motion trajectory calculated at simulation. Drift was as severe as 1.6 cm craniocaudally and 1.2 cm anteroposteriorly, which highlights the importance of continual IGRT monitoring throughout treatment. Palacios et al. (70) tracked adrenal metastases and discovered that one-third of the time anatomy positioning violated OAR or target dose constraints. van Sornsen de Koste et al. (71) followed lung, adrenal, and pancreatic tumor GTVs with 2D-cine MRI. In 90% of cases these tumors oscillated no more than 6 mm anteroposteriorly and 9 mm craniocaudally. Mean coverage was better than 94% of the GTV volume for all three tumor types (coverage was defined as a 3 mm isotropic GTV expansion).

ART discussions encompass many other considerations beyond the scope of this paper, but we highlight one more: both US commercial hybrid MRL systems use what Heukelom et al. (5) define as “serial ART” (i.e., daily images are registered to a planning scan serially without interval dose accumulation) but can conceivably be utilized for “triggered ART” (when fixed interval re-planning offline occurs) or “cascade ART” (when serial deformed dose is integrated from prior treatments). Consequently, a need for new ways of visualizing and reporting dose and morphometric alterations will soon arise. Centers that lack MRL machines but are interested in MRIgRT for abdominal cancers may find the workflow outlined in Heerkens et al. (72) informative. This phase I trial demonstrated a favorable toxicity profile (no treatment-attributable grade 3 acute or late toxicities) in 20 patients with unresectable pancreatic cancer who received 24 Gy/3 fx SBRT planned with multiparametric MRI sequences and sagittal cine MRI.

## Radiomics Standardization: A Pressing Informatics Challenge

The use of imaging biomarkers for diagnosis and prognosis is the field known as radiomics (73), or radiogenomics if the biomarkers are both radiomic and genomic (74, 75). MRI radiomics features have predicted tumor histopathology (76, 77), improved region-of-interest (ROI) auto-segmentation (78), automated radiotherapy planning (79) and predicted outcomes (e.g., survival, toxicity) (80–82). However, standardization of radiomics feature parameters is needed across radiation oncology, radiology, and nuclear medicine disciplines (83). In a systematic review of MRI radiomics applications, Jethanandani et al. concluded that MRI radiomics studies suffer from lack of standardization at multiple stages of image acquisition and processing, including MRI scanner sequence, scanner vendor, and scan acquisition parameters. There is currently no way to reliably compare between MRI radiomics studies (84). MRI has not nearly enjoyed the attention given to CT and PET radiomics standardization. Traverso et al. (85) systematically reviewed studies that assessed the repeatability and reproducibility radiomics features, finding only 1/41 papers (86) that investigated MRI.

Radiomics models should be commissioned from their ideation with a clinical decision support use case in mind (87). Studies designed to maximize the likelihood of a statistically significant finding at the expense of clinical generalizability ignore that practical implementation is a greater obstacle than discovery. To illustrate, one study that discriminated triple-negative from other breast cancer types using radiomics features ostensibly aspires to be a diagnostic alternative to biopsy (88), but would need to be less expensive yet no less accurate—a steep challenge.

Radiomics feature stability should be benchmarked on public, multi-institutional datasets (85, 89). For example, Bakas et al. (90) publicly provided radiomics features manually extracted from neuroradiologist segmentations of glioblastomas and low-grade gliomas for benchmarking future studies of these cancers. Stability should be benchmarked per anatomic site, since features that are repeatable and reproduceable at one site may degrade in the context of another.

## Initiatives for FAIRer Data

Inter-institutional findable, accessible, interoperable, reusable (FAIR) (91) and high-quality data is essential for establishing the clinical value of MRIgRT. However, political, financial, and legal obstacles silo data within institutions (92) and ethical questions surrounding health data analytics, particularly by tech institutions currently not subject to the same patient privacy laws as healthcare institutions, are unresolved (93, 94). The need for FAIR data is not exclusive to MRIgRT: FAIR data are critical for achieving the vision for machine learning in healthcare widely (95–97).

A recent AAPM council observed that RT data increase as cancer patients survive longer and genomic data move toward mainstream clinical use (98). The council predicted, “Whereas success in medical research in the past has favored very large single institutions that can develop a critical mass of knowledge and resources in close physical proximity, *diffuse networks of institutions able to generate and share information will have an advantage in the future*” (emphasis added). We now conclude with a discussion of two emerging initiatives for FAIRer data: “distributed learning,” a method for inter-institutional machine learning, and Fast Healthcare Interoperability Resources (FHIR, pronounced “fire”) a healthcare data standard.

### Distributed Learning

Distributed learning refers to training machine learning models on multi-institutional data without sharing the data (99–101). The key is that the statistical weights and parameters of the machine learning model travel between institutions, not the data. Distributed learning is an option method for generating statistical models for emerging technologies, such as MRLs (7, 8). Distributed learning is possible between horizontally-partitioned data (same features, different patients) or vertically-partitioned data (different features, same patients) (101, 102).

### FHIR

Conceived in 2014, FHIR is a specification for health data formatting (i.e., XML and JSON) and messaging (i.e., RESTful application programming interfaces). FHIR-conforming data are retrievable between health information technology softwares (103), and FHIR may soon be a mandated EHR specification (104). FHIR provides a standard for storing and querying radiotherapy data objects independent of vendor, such as total dose, or DICOM-RT structure sets. Conformity with FHIR also makes it possible to build applications that integrate health information technologies. For example, Substitutable Medical Applications and Reusable Technologies (SMART) is an EHR app platform built on FHIR (105). SMART delineates authorization, authentication, and user interface specifications for FHIR-conforming apps. Because RT treatment planning and information systems are usually separate from EHRs (98, 106), initiatives like SMART on FHIR envision a future where it is possible to build RT task-specific apps into EHRs (107). Open-source, FHIR-conforming applications may be one platform for scaling MRIgRT software.

## Author Contributions

KK and CF proposed the idea for this manuscript. KK drafted the manuscript. KK, CF, JW, and BS iteratively revised the manuscript.

### Conflict of Interest

CF has received direct industry grant support, speaking honoraria, and travel funding from Elekta AB. BS has intellectual property licensed to Oncora Medical. The remaining authors declare that the research was conducted in the absence of any commercial or financial relationships that could be construed as a potential conflict of interest.
